# HIV Self-Testing Could “Revolutionize Testing in South Africa, but It Has Got to Be Done Properly”: Perceptions of Key Stakeholders

**DOI:** 10.1371/journal.pone.0122783

**Published:** 2015-03-31

**Authors:** Tawanda Makusha, Lucia Knight, Miriam Taegtmeyer, Olivia Tulloch, Adlai Davids, Jeanette Lim, Roger Peck, Heidi van Rooyen

**Affiliations:** 1 Human Sciences Research Council, Durban, South Africa; 2 Liverpool School of Tropical Medicine, Liverpool, United Kingdom; 3 School of Public Health, University of Western Cape, Cape Town, South Africa; 4 PATH, Seattle, Washington, United States of America; University of Washington, UNITED STATES

## Abstract

South Africa bears the world’s largest burden of HIV with over 6.4 million people living with the virus. The South African government’s response to HIV has yielded remarkable results in recent years; over 13 million South Africans tested in a 2012 campaign and over 2 million people are on antiretroviral treatment. However, with an HIV & AIDS and STI National Strategic Plan aiming to get 80 percent of the population to know their HIV status by 2016, activists and public health policy makers argue that non-invasive HIV self-testing should be incorporated into the country HIV Counseling and Testing [HCT] portfolios. In-depth qualitative interviews (N = 12) with key stakeholders were conducted from June to July 2013 in South Africa. These included two government officials, four non-governmental stakeholders, two donors, three academic researchers, and one international stakeholder. All stakeholders were involved in HIV prevention and treatment and influenced HCT policy and research in South Africa and beyond. The interviews explored: interest in HIV self-testing; potential distribution channels for HIV self-tests to target groups; perception of requirements for diagnostic technologies that would be most amenable to HIV self-testing and opinions on barriers and opportunities for HIV-linkage to care after receiving positive test results. While there is currently no HIV self-testing policy in South Africa, and several barriers exist, participants in the study expressed enthusiasm and willingness for scale-up and urgent need for further research, planning, establishment of HIV Self-testing policy and programming to complement existing facility-based and community-based HIV testing systems. Introduction of HIV self-testing could have far-reaching positive effects on holistic HIV testing uptake, giving people autonomy to decide which approach they want to use for HIV testing, early diagnosis, treatment and care for HIV particularly among hard-to reach groups, including men.

## Background

South Africa bears the world’s largest burden of HIV with over 6.4 million people living with the virus [1]. The South African government’s response to HIV has yielded remarkable results in recent years, over 42% of South Africans tested for HIV, with 52% of females and 37% of males testing in 2012 [1]. While HIV counseling and testing (HCT) coverage has increased in the country there is a need for continued expansion of HCT, coupled with linkage to care, as only 31% of known HIV positive persons were on antiretroviral treatment (ART) [1]. People living with HIV (PLHIV) are often diagnosed late, resulting in avoidable morbidity, mortality, and transmission of the virus [2–4].

Effective control of the HIV epidemic in South Africa requires high coverage of HIV testing and linkage to treatment amongst all groups [2, 3, 5, 6]. Despite improved testing coverage in the general population, hard-to-reach groups—such as men who have sex with men (MSM), commercial sex workers (CSW), adolescents, and all men—are underserved, with poor access to testing and treatment services, high levels of stigma and discrimination and poor integration into health systems [7].

Facility-based HCT alone (whether client-or provider-initiated) will not be sufficient to achieve universal access to HIV testing and treatment. Barriers to testing through standard facility-based options are complex and varied, and include inconvenient clinic hours, long distance from facilities and the cost of traveling to clinics [8]. Concerns regarding stigma, discrimination and the fear of positive results continue to limit uptake of HCT in many high HIV prevalence settings, including South Africa [5, 9]. Compelling evidence is emerging in South Africa and other contexts that community-based models of testing (mobile and home-based) are capable of reaching a wider range of target groups, expanding the geographic coverage and uptake of HCT and addressing some of the barriers to more conventional HCT models such as time, cost and distance [10–15].

Based on increasing evidence from feasibility and acceptability studies in some parts of Africa, activists and public health policy makers argue that non-invasive HIV self-testing (HIVST)—an HIV test collected, performed and interpreted in private by the individual who wants to know their HIV status [16]—should be incorporated into the country’s HCT portfolios. The view is that like other community-based models, HIVST has the potential to bypass facility-based barriers by offering a convenient, confidential, and unsupervised HIV testing option for groups not currently accessing HIV testing [8, 17, 18], possibly encouraging earlier linkage to treatment and care services [19, 20].

HIV self-testing is not without challenges. The most significant argument against HIVST relates to concerns about the absence of in-person pre- and post-test counseling which have been the hallmark of traditional voluntary or client-centred HCT [3]. Concerns are that the absence of counseling could increase the risk of distress and suicide for those who test HIV-positive [21], lead to coerced testing, increase the possibility of social harms to more vulnerable individuals [22], lower rates of linkages to treatment and care, and result in missed opportunities for prevention in high risk negatives. However, a recent review of 300 articles showed little evidence of psychological, medical or social harms through selected self-tests as well as HIV self-tests [23].

While the opportunities and barriers afforded by HIVST have been explored in several settings including the United States, Singapore, Kenya and Malawi [10, 20, 24, 25], perceptions of HIVST from South Africa, a country severely impacted by HIV, are less established. Several discussion and opinion pieces on HIVST in the South African context exist [21, 26, 27], but there are few empirical reports on the perceptions and attitudes of South African stakeholders’ on the constraints and opportunities of introducing HIVST [8, 28]. This paper presents a unique perspective of various stakeholders with influence in HIV prevention and treatment regarding the requirements for the introduction of HIVST in South Africa. It does so by reporting on the qualitative component of a larger study which assessed the feasibility and usability of a number of HIVST prototypes among lay users in Kenya, Malawi and South Africa, findings from which are reported elsewhere [29].

## Methodology

### Sampling

In-depth qualitative interviews (N = 12) with key stakeholders were conducted from June to August 2013 in South Africa. These included two government officials, four non-governmental (NGO) stakeholders, two donors, three academic researchers, and one international stakeholder. Key informants were national decision-makers in HIV programming, HIV test procurement, policy development and researchers in South Africa and beyond. It was important for us to target these specific stakeholders because of their influence in HIV prevention and treatment research and policy formulation. We used a purposive sampling approach to capture a range of attitudes, opinions and experiences regarding HIVST in South Africa. Snowball sampling was also used to source additional participants for interviews [30]. Stakeholders were contacted via e-mail or telephonically and interviews were scheduled at their convenience. Interviews were conducted in person or over the telephone.

### Data collection and analysis

Study results were not meant to be generalizable, but to capture a diversity of opinions, experiences and attitudes towards HIVST. Respondents were interviewed on their interest in HIVST; potential distribution channels for HIVST kits to target groups; perception of requirements for diagnostic technologies that would be most amenable to HIVST and opinions on barriers and opportunities for HIV-linkage to care after receiving positive test results. All interviews were conducted in English by trained qualitative researchers using a semi-structured interview guide. Interviews were audio-recorded and transcribed for analysis. Investigators worked iteratively, reading individual transcripts and agreeing on a coding framework. The first author, applied descriptive or topical codes to the data according to the coding framework using *Nvivo 10* software [31].

All 12 transcripts’ codes were revised and compared using the constant comparison method [32]. Emerging themes regarding the opportunities and barriers to the introduction of HIVST in South Africa were noted and the account of opportunities and barriers to HIVST that emerged from the data was developed. The researchers reviewed the data several times to verify themes and explanations and ensure that interpretation of the data was an accurate account of what respondents said [33]. Transcripts were also reviewed by an independent external expert and wider discussions comparing emerging themes in Malawi and Kenya gave new insights to themes here. Each step of the analysis was checked by returning to relevant data extracts from each interview, therefore employing comprehensive data treatment [32].

### Ethical considerations

Ethical approval for the study was obtained from the Research Ethics Committee of the Human Sciences Research Council (HSRC), South Africa. Internationally accepted ethical standards for conducting research were observed, which include getting written informed consent from all participants, ensuring that they had been briefed about and understood what the research involved.

## Results

Levels of enthusiasm for HIVST were high amongst stakeholders with the view that: “… it [could] revolutionize testing in South Africa” (Academic 2); it was timely: “This is something that is long overdue” (Academic 3) and that it could be used to achieve universal testing: “Every South African should be tested at least once a year…we need to have various approaches of reaching people, innovative and yet user friendly” (Government representative 1). We expand on stakeholder perceptions on the opportunities and barriers to HIVST in the sections that follow.

### Opportunities

All stakeholders felt that HIVST presented the country with several unique opportunities: it encouraged individual autonomy; it allowed for confidentiality and privacy in testing; it had the potential to reach those missed by current HCT approaches and it could eliminate stigma.

#### Self-testing: autonomy, privacy and confidentiality

For several participants, the most positive aspect of HIVST was its potential to increase the autonomy of users by putting them in charge of their health and their bodies.

*HIV self-testing will provide opportunities for people to be in charge of their own lives and take responsibility of their own bodies*. *They want to know their HIV status without a third party involved*. *They want to be in total control of the process (NGO representative 3)*.

For several stakeholders autonomy was closely tied to the confidentiality afforded by HIVST:

*So I think the benefits also are pure confidentiality*, *if I can own the process myself*, *you know I would have that confidential aspect of HIV doing it—the empowerment to take responsibility for my life because if I can go as far as to decide that “You know what*, *I need to be testing myself at this level”*, *it means I am taking responsibility for my sexual health… [and] I am going to think about it in light of how I manage my life (Academic 2)*.

#### HIVST could identify hidden populations, especially men

Stakeholders felt that HIVST could have particular value for hidden, hard-to-reach populations who are currently not accessing HCT.

*So*, *I think if we look at the international literature on self-testing*, *people who are unlikely to use health facilities because of perhaps perceived bias against them might be more likely to use them*. *Whatever that group is; sex workers*, *MSM*. *Whatever that sub-group is that feels they are not going to get the best care in recurrent health facilities might feel self-testing is a way of getting that information without facing the questions and attitudes from staff… I think it has some potential to reach some difficult groups to reach; maybe men are more likely to use it than women because their health seeking behaviour is different*. *Maybe it will find groups that are currently reluctant to test (Academic 2)*.

Of these hard-to-reach populations, most stakeholders felt that HIVST would be an ideal option for men, who place greater premium on privacy and convenience than others:

*We have been testing for the past 12 years*, *and talking to people who come and get tested with us*. *Talking to men about testing… and the things they wanted is the thing this product [HIVST] provides*, *which is privacy and confidentiality*. *They don’t want to sit there with somebody; they want to do it themselves (NGO representative 4)*.

#### HIVST has the capacity to mitigate stigma

Stakeholders felt that HIVST has the capacity to normalize HIV testing and this could lead to HIV being treated like any other chronic, but manageable, condition. Collectively, these conditions could impact stigma and discrimination.

*Others have said it [HIVST] will alleviate the clinic stuff*, *queues*, *going to a centre or a mobile clinic where people are saying “Oh*, *you are going for an HIV test”*, *therefore there is an assumption made that you may be positive*. *So in other words*, *the culture of stigma still sits there (NGO representative 3)*.

### Barriers

The barriers to the introduction of HIVST in South Africa included: lack of counseling; difficulty in ensuring linkages to care; potential abuse of human rights; and quality and accuracy of the tests.

#### Lack of counseling

One of the major concerns expressed, particularly by government and donor representatives, was the fact that face-to-face counseling and support were missing from the HIVST. These are viewed as essential components of all current testing models in South Africa:

*But most important… we need to ensure there will be constant access to counseling*. *We should not undermine the value of counseling*. *That is why whenever we talk about HIV testing*, *or whatever the case might be*, *we talk HIV counseling and testing…You will never find HIV testing that does not have the C*, *so the C for me is a crucial part (Donor 2)*.

Stakeholders were adamant about the provision of a toll-free counseling line, including online and mobile (mHealth) services to accompany HIVST. However one respondent expressed serious concerns about the poor quality of the existing HIV helpline, and another thought the helpline services may simply direct callers to the nearest HIV clinic rather than providing full counseling.

#### Difficulty in ensuring linkages to care among those with positive results

Another barrier to HIVST, noted by all participants, is that it does not provide a personal referral mechanism to link HIV-positive people to treatment and care. Although it is acknowledged that linkage to care is a challenge for both facility- and community-based HCT approaches, in the HIVST model this issue may be exacerbated because the process is not mediated by a health care worker or lay counselor who could provide, or encourage linkage to care and support for those who test HIV-positive.

Most stakeholders felt that a way to address this barrier could be to ensure that HIVST is introduced as a screening test—just like a home pregnancy test, requiring that people still go to health facilities for confirmation of an HIV diagnosis and possible linkage to care. Participants also reiterated the importance of having clear instructions directing people to the relevant linkages to care and support services in their area on the HIVST kits and having a 24 hour-7 day a week toll-free number, including online and mHealth services.

*…the product…at the very least should be a brochure with contact details for care and support*. *At the very least there must be that so that the person can at least call a toll free number or make contact with someone via online or mHealth services*. *Not all people will contact the toll free number or contact someone but at the very least I think that is important (NGO representative 2)*.

#### Potential abuse of human rights

Several participants felt there was the potential for abuse or misuse of a HIVST or for an increase in negative consequences such as increased cases of suicide in response to HIV positive test results.

*There is always a downside to it but we always hope that …*. *by self-testing you will encourage your partner*, *or your family to get tested but this could be totally opposite*. *We know that in South Africa there is higher degree of domestic violence for example*, *partner violence so yes you are right*. *I think it’s that balance that you can acknowledge the benefits while also looking at potential risks and the extent to which those risks such as partner violence*, *gender violence and abuse of authority will outweigh the advantages of expanded self-testing (Academic 3)*.

#### Quality assurance

A few stakeholders expressed concerns about the quality and accuracy of the actual tests and the ability of lay users to conduct the tests, although overall there was relatively high confidence in rapid test accuracy. Lower sensitivity was seen as an acceptable public health risk provided that there were overall public health gains through new diagnoses from HIVST.

*Well*, *I think the other challenge as well depends on how much sensitivity would you want to lose on a test*, *because in quality control the testing is going to be a challenge*. *There might be settings where the test is not used properly… But there will still be some loss of sensitivity but if we agree that sensitivity of 90% of 80% is acceptable to us because we have an efficient system which allows people to enter into our care and treatment programme that’s fine*. *We are not really bothered about it*. *But if our sensitivity really drops below our critical value then what’s the purpose (Academic 1)*.

Some respondents felt that the introduction of HIVST could exacerbate the challenges already experienced with quality assurance in the facility-based HCT system.

*We are having quality assurance challenges already and with self-testing it would even highly increase*. *The problem is that you do not have a quality control at hand*, *yet as a diagnostic test you need to have a quality control to see whether you applied the test correctly therefore this is difficult to monitor when you conduct a self-test (Academic 3)*.

### Requirements for the Introduction of HIVST

In addition to these barriers, stakeholders highlighted several other conditions that would have to be addressed before scale-up of HIVST. Although there have been marked developments in obtaining pre-market approval for diagnostic technologies in South Africa, such as the Medicines and Related Substances Amendment Act of 2008 (not yet promulgated); the draft regulations issued in terms of that Act in April 2014; and the draft guidelines published for comment by the Medicines Control Council in September 2014, the country does not currently have a proper regulatory system to govern the wide-scale introduction of HIVST [16, 34], including other diagnostic technologies. Several participants felt that this was a key issue that had to be addressed.

*South Africa needs to address a number of issues in terms of its laws and policies on medicines and related substances*. *How will we ensure quality assurance and ensure that the manufacturers are not false advertising*? *How will we ensure that the self-tests are manufactured by an accredited facility*? *All these issues need to be addressed to provide a good regulatory system (Academic 1)*.

Stakeholders reiterated that for HIVST to work, it has “got to be done properly”. This embraced the need for extensive planning and widespread consultations before the introduction of HIVST in South Africa.

*I sense that there are a lot of questions that need to be answered on whether we are ready*, *who are we targeting this for*, *and for what purpose and how much gain there [will] be to self-testing*. *I know there is this big drive*, *I know home testing campaign has grown*, *all sort of initiatives*, *informal settings have facilities*. *The question is when we want to expand that*, *who do we really want to target*? *(Government representative 2)*

Another area which requires extensive consultation before the introduction of HIVST related to the costs of the test, and who should bear these costs. While most participants argued that the HIVST kits should be available for free, some stated that they should be sold at a subsidized price. There was no consensus on who should pay for the HIVST kits, with some participants saying that government should pay for the tests, while others argued that government, donors and NGOs should subsidize the HIVST kits and they should be available for free to the general population, in public and private health care facilities, in schools and in workplaces. However, what was clear from all these discussions was that the HIVST kits should be affordable to everyone who wants to access them.

Participants also highlighted the importance of setting up a proper and efficient monitoring and evaluation programme for HIVST. Participants noted the importance of setting up effective strategies for measuring HIV incidence and prevalence; and the extent to which people who have conducted the HIVST report their status.

## Discussion

This qualitative study describes the perceptions of key stakeholders who influence policy on HIV testing, about South African readiness for HIVST. Notwithstanding the potential barriers that might come with the adoption of HIVST, the results indicate significant support for the introduction of HIVST in South Africa from various stakeholders who influence HIV prevention and treatment research and policy formulation. This is, to our knowledge, a unique contribution to the emerging literature on HIVST in this high-prevalence context.

Similar to other studies, our results indicate that if HIVST is to be implemented in South Africa, measures should be taken to ensure that poor, marginalized and hard-to-reach populations, are able to access the service by making the HIVST kits affordable for the majority [35]. This study goes a step further to highlight that for successful adoption and implementation of HIVST in South Africa, policy making needs to be strategically aligned to market forces, industry, advocacy and public demand for the HIVST. Respondents argued that for South Africa to establish a strong, inclusive HIVST programme, the government, international donor organizations and non-governmental organizations should subsidize the costs of the HIVST kits ensuring all have access.

Men and women make unequal use of public health facilities in many parts of Africa, with women having more contact with health facilities, mainly through reproductive and child health services [1]. Further, health services are not male-friendly spaces, with operating hours that often clash with work obligations, and provider attitudes that may lack sensitivity to men’s needs, further alienating them [6, 15]. As a result of this gender disparity in health care, men have fewer opportunities and disproportionately poorer access to HIV prevention, care, and treatment services. Our results suggest that HIVST has the potential to address some of these gender disparities in testing and treatment, by affording men the opportunity to act autonomously, to make decisions for themselves and their health privately and confidentially, on their own terms, and at their convenience [36].

Although not unique to HIVST, the optimism and opportunities presented by HIVST were tempered by the caution that for HIVST to work several concerns must be addressed. Key amongst these is the lack of in-person counseling and the potential for harm that may result because of this [22, 25, 27]. Like others, South African stakeholders also lamented the limited opportunities for linkage to care in the HIVST model [26]. Results from our study corroborate previous reviews stating that linking HIV positive people to treatment and care is an on-going problem, even in facility-based testing centres [2, 22, 26]. Respondents emphasized that linkages to treatment and care depend on individual will. Our study points to several strategies for addressing these issues. First, as per the WHO guidelines [4], using HIVST as a screening test would encourage confirmatory testing through a health facility and may facilitate linkage to care.

Second, our study found that the lack of a face to face counselor provides a unique opportunity for researching innovative strategies to provide counseling (i.e. through online or mHealth technologies, via a toll free number) and to link people to care. Self-testing instruction kits could also provide much needed information on what HIV positive people should do after they test, namely; where to get further counseling, where to go for confirmatory tests and CD4 counts, how to get linked to treatment, care and support.

Concerns that HIVST may lead to coercion in the home environment, creating situations where the individual rights of women and children are abused have been raised by these stakeholders and elsewhere [22, 23]. However, throughout the growing literature on HIVST, there is no data to support this claim in the South African context [27]. Based on the stakeholders’ views and other previous studies, this paper recommends that HIVST should not be restricted based on fears of harm, but rather that as HIVST is expanded, researchers and policy makers should pay particular attention to monitoring and measuring of unintended harm [23].

To mitigate the unintended harm and the potential abuse of individual rights and forced HIV testing in the HIVST model, this study emphasizes the importance of establishing a proper regulatory system that protects children, women and employees from being tested against their will. As highlighted in previous studies, our study revealed that the national health policies which fall within the confines of medical device regulation, should guide and inform HIVST implementation in both the public and private sectors [34].

One limitation to the study was that we were not really able to disaggregate findings by type or level of key informant. For example, it was difficult to establish whether donors felt differently from government officials on the introduction of HIVST in South Africa.

## Conclusions

While there is currently no HIVST policy in South Africa [16, 34], and several barriers exist, there is willingness for scale-up and urgent need for further research, planning, establishment of HIVST policy and programming to complement existing facility-based and community-based HIV testing systems. Our study suggests that the introduction of HIVST could have great potential and far-reaching positive effects on holistic HIV testing uptake, early diagnosis, treatment and care for HIV particularly among hard-to reach groups, including men. In a country where the HIV & AIDS and STI National Strategic Plan aims to eliminate HIV incidence by 50 per cent and get 80 per cent of the population knowing their HIV status by 2016 [37], it is time to give people the autonomy to decide which approach they want to use for HIV testing.

## References

[1] Shisana O, Rehle T, Simbayi L, Zuma K, Jooste S, N. Z, et al South African National HIV Prevalence, Incidence and Behaviour Survey, 2012. Cape Town: Human Sciences Research Council, 2014.

[2] WHO. Report on the first international symposium on self-testing for HIV: the legal, ethical, gender, human rights and public health implications of HIV self-testing scale-up. Geneva, Switzerland: 2013.

[3] WHO. Towards universal access: scaling up priority HIV/AIDS interventions in the health sector, progress report 2010 Geneva: WHO, 2010.

[4] WHO, UNICEF, UNAIDS. Global HIV/AIDS response Epidemic update and health sector progress towards universal access. Progress report. Geneva, Switzerland: WHO UNICEF, UNAIDS, 2011.

[5] Spielberg F, Levine RO, Weaver M. Self-testing for HIV: a new option for HIV prevention? The Lancet infectious diseases. 2004;4(10):640–6. 15451493 10.1016/S1473-3099(04)01150-8

[6] van Rooyen H, Richter L, Coates T, Boettiger M. Approaches to HIV Counselling and Testing: Strengths and Weaknesses, and Challenges for the Way Forward In: Rohleder P, Swartz L, Kalichman SC, Simbayi LC, editors. HIV/AIDS in South Africa 25 Years On. New York: Springer 2009 p. 165–82.

[7] Baral S, Burrell E, Scheibe A, Brown B, Beyrer C, Bekker LG. HIV risk and associations of HIV infection among men who have sex with men in peri-urban Cape Town, South Africa. BMC Public Health. 2011;11(1):766. 21975248 10.1186/1471-2458-11-766 PMC3196714

[8] Pant Pai N, Behlim T, Abrahams L, Vadnais C, Shivkumar S, Pillay S, et al Will an Unsupervised Self-Testing Strategy for HIV Work in Health Care Workers of South Africa? A Cross Sectional Pilot Feasibility Study. PLoS ONE. 2013;8(11):e79772 10.1371/journal.pone.0079772 24312185 PMC3842310

[9] Kalichman SC, Simbayi LC. HIV testing attitudes, AIDS stigma, and voluntary HIV counselling and testing in a black township in Cape Town, South Africa. Sexually Transmitted Infections. 2003;79(6):442–7. 14663117 10.1136/sti.79.6.442 PMC1744787

[10] Choko A, Desmond N, Webb E, Chavula K, Napierala-Mavedzenge S, Gaydos C, et al The uptake and accuracy of oral kits for HIV self-testing in high HIV prevalence setting: A cross-sectional feasibility study in Blantyre, Malawi. PLoS medicine. 2011;8(10). 10.1371/journal.pmed.1001111 21990966 PMC3186813

[11] Frith L. HIV self-testing: a time to revise current policy. Lancet. 2007;369(9557):243–5. 17240291 10.1016/S0140-6736(07)60113-5

[12] Ganguli I, Bassett I, Dong K, Walensky R. Home testing for HIV infection in resource-limited settings. Curr HIV/AIDS Rep. 2009;6(4):217–23. 19849965 10.1007/s11904-009-0029-5 PMC3523205

[13] Merson MH, Feldman EA, Bayer R, Stryker J. Rapid self testing for HIV infection. The Lancet. 1997;349(9048):352–3. 9024392 10.1016/S0140-6736(96)08260-8

[14] Wright AA, Katz IT. Home testing for HIV. New England Journal of Medicine. 2006;354(5):437–40. 16452553 10.1056/NEJMp058302

[15] van Rooyen H, McGrath N, Chirowodza A, Joseph P, Fiamma A, Gray G, et al Mobile VCT: Reaching Men and Young People in Urban and Rural South African Pilot Studies (NIMH Project Accept, HPTN 043). AIDS Behav. 2013;17(9):2946–53. 10.1007/s10461-012-0368-x 23142856 PMC3597746

[16] Southern African AIDS Trust. Multi-Jurisdiction review of the legal issues surrounding the distribution of HIV self-testing kits Johannesburg: Southern African AIDS Trust, 2014.

[17] Myers JE, El-sadr WM, Zerbe A, Branson BM. Rapid HIV self-testing: long in coming but opportunities beckon. AIDS. 2013;Publish Ahead of Print:10.1097/QAD.0b013e32835fd7a0. 10.1097/QAD.0b013e32835fd7a0 23807269

[18] Krause J, Subklew-Sehume F, Kenyon C, Colebunders R. Acceptability of HIV self-testing: a systematic literature review. BMC public health. 2013;13:735 10.1186/1471-2458-13-735 23924387 PMC3750621

[19] Lugada E, Millar D, Haskew J, Grabowsky M, Garg N, Vestergaard M, et al Rapid implementation of an integrated large-scale HIV counseling and testing, malaria, and diarrhea prevention campaign in rural Kenya. PLoS One. 2010;5(8):12435. 10.1371/journal.pone.0012435 PMC2928737 20865049

[20] Negin J, Wariero J, Mutuo P, Jan S, Pronyk P. Feasibility, acceptability and cost of home-based HIV testing in rural Kenya. Tropical Medicine & International Health. 2009;14(8):849–55. 19552646 10.1111/j.1365-3156.2009.02304.x

[21] Richter M, Venter WDF, Gray A. Home self-testing for HIV: AIDS exceptionalism gone wrong. SAMJ: South African Medical Journal. 2010;100:636–42. 10.7196/samj.4198 21081002

[22] Napierala Mavedzenge S, Baggaley R, Corbett EL. A Review of Self-Testing for HIV: Research and Policy Priorities in a New Era of HIV Prevention. Clinical Infectious Diseases. 2013;57(1):126–38. 10.1093/cid/cit156 23487385 PMC3669524

[23] Brown A, Djimeu E, Cameron D. A review of the evidence of harm from self-tests. AIDS Behav. 2014;18(4):445–9. 10.1007/s10461-014-0831-y PMC4094790 24989129

[24] Ferrand RA, Trigg C, Bandason T, Ndhlovu CE, Mungofa S, Nathoo K, et al Perception of Risk of Vertically Acquired HIV Infection and Acceptability of Provider-Initiated Testing and Counseling Among Adolescents in Zimbabwe. American Journal of Public Health. 2011;101(12):2325–32. 10.2105/AJPH.2011.300250 22021300 PMC3222430

[25] Kalibala S, Tun W, Muraah W, Cherutich P, Oweya E, Oluoch P. “Knowing Myself First”: Feasibility of self-testing among health workers in Kenya Nairobi: Population Council, 2011.

[26] Gardner J. HIV home testing—a problem or part of the solution?. South African Journal of Bioethics and Law. 2012;5(1):15–9.

[27] Richter ML, Venter WD, Gray A. Forum: Enabling HIV self-testing in South Africa. Southern African Journal of HIV Medicine. 2012;13(4):186–7.

[28] Dong M, Regina R, Hlongwane S, Ghebremichael M, Wilson D, Dong K. Can laypersons in high-prevalence South Africa perform an HIV self-test accurately?. 20th International AIDS Conference, 2014 July 20–25; Melbourne, Australia: International AIDS Society; 2014.

[29] Peck RB, Lim JM, van Rooyen H, Mukoma W, Chepuka L, Bansil P, et al What should the ideal HIV self-test look like? A usability study of test prototypes in unsupervised HIV self-testing in Kenya, Malawi, and South Africa. AIDS Behav. 2014;18(4):422–32. 10.1007/s10461-014-0818-8 24947852

[30] Goodman LA. Snowball sampling. The annals of mathematical statistics. 1961:148–70.

[31] Green J, Thorogood N. Qualitative Methods for Health Research. London: Sage; 2004.

[32] Silverman D. Doing Qualitative Research. 3rd ed. London: Sage; 2010.

[33] Patton MQ. Qualitative evaluation methods Newbury Park: Sage; 1980.

[34] Wong V, Johnson C, Cowan E, Rosenthal M, Peeling R, Miralles M, et al HIV Self-Testing in Resource-Limited Settings: Regulatory and Policy Considerations. AIDS Behav. 2014;18(4):415–21. 10.1007/s10461-014-0825-9 24957979

[35] Ng OT, Chow AL, Lee VJ, Chen MI, Win MK, Tan HH, et al Accuracy and user-acceptability of HIV self-testing using an oral fluid-based HIV rapid test. PLOS ONE. 2012;7(9). 10.1371/journal.pone.0045168 PMC3444491 23028822

[36] Kumwenda M, Munthali A, Phiri M, Mwale D, Gutteberg T, MacPherson E, et al Factors shaping initial decision-making to self-test amongst cohabiting couples in urban Blantyre, Malawi. AIDS Behav. 2014 18(4):396–404. 10.1007/s10461-014-0817-9 PMC4102820 24929834

[37] South African National AIDS Council. HIV & AIDS and STI National Strategic Plan 2012–2016. Pretoria: South African Government, 2011.

